# The pathohistological subtype strongly predicts survival in patients with ampullary carcinoma

**DOI:** 10.1038/s41598-019-49179-w

**Published:** 2019-09-03

**Authors:** Carolin Zimmermann, Steffen Wolk, Daniela E. Aust, Frieder Meier, Hans-Detlev Saeger, Florian Ehehalt, Jürgen Weitz, Thilo Welsch, Marius Distler

**Affiliations:** 1 Department of Visceral, Thoracic and Vascular Surgery, University Hospital Carl Gustav Carus, Technische Universität Dresden, Fetscherstr. 74, 01307 Dresden, Germany; 2 Institute for Pathology, University Hospital Carl Gustav Carus, Technische Universität Dresden, Fetscherstr. 74, 01307 Dresden, Germany; 3 Tumor and Normal Tissue Bank of the UCC/NCT Site Dresden, University Hospital Carl Gustav Carus, Technische Universität Dresden, Fetscherstr. 74, 01307 Dresden, Germany

**Keywords:** Pancreatic cancer, Surgical oncology

## Abstract

Ampullary cancer represents approximately 6% of the malignant periampullary tumors. An early occurrence of symptoms leads to a 5-year survival rate after curative surgery of 30 to 67%. In addition to the tumor stage, the immunohistological subtypes appear to be important for postoperative prognosis. The aim of this study was to analyze the different subtypes regarding their prognostic relevance. A total of 170 patients with ampullary cancer were retrospectively analyzed between 1999 until 2016 after pancreatic resection. Patients were grouped according to their pathohistological subtype of ampullary cancer (pancreatobiliary, intestinal, mixed). Characteristics among the groups were analyzed using univariate and multivariate models. Survival probability was analyzed by the Kaplan-Meier method. An exact subtyping was possible in 119 patients. A pancreatobiliary subtype was diagnosed in 69 patients (58%), intestinal in 41 patients (34.5%), and a mixed subtype in 9 patients (7.6%). Survival analysis showed a significantly worse 5-year survival rate for the pancreatobiliary subtype compared with the intestinal subtype (27.5% versus 61%, *p* < 0.001). The mean overall survival of patients with pancreatobiliary, intestinal, and mixed subtype was 52.5, 115 and 94.7 months, respectively (*p* < 0.001). The pathohistological subtypes of ampullary cancer allows a prediction of the postoperative prognosis.

## Introduction

Ampullary cancer is a rare malignant disease, occurring in approximately 0.2% of all gastrointestinal tumors^1–3^. Its localization characteristic symptoms like jaundice and pain usually occur earlier compared with other malignant pancreatobiliary tumors like pancreatic cancer^3^. This is possibly the reason for the better prognosis of ampullary carcinomas with a reported 5-year survival rate of 45% in resected patients^4,5^. For diagnosis, computed tomography, esophagogastroduonoscopy, endoscopic guided biopsy, endosonography, endosonography-guided biopsy, and ultrasound are useful. The current gold standard of treatment is the oncological resection of the tumor together with lymph node dissection. For this reason, in most cases a pancreatic head resection is necessary. Different studies have shown that the lymph node status in ampullary cacinomas is an important prognostic predictor^6^. The use of adjuvant chemotherapy in patients with resected ampullary cancers has not been proven in prospective randomized clinical trials yet. Furthermore, multiple studies have shown no benefit in overall survival or disease-free survival for adjuvant treatment^7^. According to the histopathologic characteristics, different subtypes of ampullary carcinomas are described^8,9^. The pancreatobiliary subtype arises from simple mucinous epithelium of the distal common bile duct, the distal pancreatic duct, or common ampullary duct with simple or branching glands and small solid cell-nests enclosed by desmoplastic stroma^10–12^. This epithelium builds the mucosal lining of the ampulla^11,12^. One theory is that the degeneration of the epithelium could follow an analogous dysplasia-adenocarcinoma sequence akin to pancreatic intraepithelial neoplasia^10^. From the intestinal mucosa, which covers the papilla, originates the intestinal type with well-formed tubular glands, complex cribriform areas, and solid nests^13^. This epithelium might be arise through an adenoma-dysplasia-adenocarcinoma sequence related to colon cancer^14^. The mixed subtype is described as a tumor consisting of more than 25% of each differentiation or as a tumor consisting of hybrid differentiation like intestinal architecture with pancreatobiliary cytology^15^. When comparing the subtypes, they differ in prognosis with 5-year survival rates ranging from 20% for the pancreatobiliary subtype to 88% for the intestinal subtype for resected patients^4^. Therefore, there is reason to believe that the prognosis of papillary carcinoma is related to the subtype^11^. Compared with other studies more patients were included in the analysis to validate the hypothesis that the pathohistological subtype predicts survival in patients with resected ampullary cancer.

## Results

### Patient cohort and operation characteristics

We evaluated 170 patients with ampullary cancer, who underwent resection between 1999 and 2016 at our department. A total of 119 from these 170 patients with complete immunohistochemical subtype analysis were included in this study. By immunohistochemical analysis of the surgical specimen, we identified the pancreatobiliary subtype in 69 patients (58%), the intestinal subtype in 41 patients (34.5%), and the mixed subtype in 9 patients (7.5%). Table 1 lists the clinical and pathological characteristics according to the morphological subtypes of the patients with ampullary carcinoma.

**Table 1:** Clinicopathological characteristics.

	Pancreatobiliary	Intestinal	Mixed	All subtypes
No patients	69 (58%)	41 (34.5%)	9 (7.5%)	119
Sex (m/w)	42/27	26/15	5-Apr	73/47
**Age** (**years**)				
mean ± SD	68.04 ± 10.4	65.4 ± 9.7	71.0 ± 8.6	67.4 ± 10.0
**CEA ng/mL**				
mean ± SD	7.6 ± 29	2.2 ± 2.08	1.3 ± 0.86	5.3 ± 22.1
**CA 19-9 U/mL**				
mean ± SD	373.2 ± 1019	79.5 ± 259.9	213.3 ± 382.5	256.4 ± 799.6
**Bilirubin µmol/l**				
mean ± SD	68.2 ± 95.4	71.0 ± 101.3	49.6 ± 111.0	67.7 ± 97.9
Pre-operative diabetes	22 (31.9%)	11 (26.8%)	2 (22.2%)	35 (29.4%)
Pain	23 (33.3%)	14 (34.1%)	5 (55.6%)	42 (35.3%)
Obstructive jaundice	52 (75.4%)	28 (68.3%)	6 (66.7%)	86 (72.3%)
Weight loss	42 (60.9%)	20 (48.8%)	4 (44.4%)	66 (55.5%)
**Tumor size** (**mm**)				
Mean ± SD	25.5 ± 13.8	24.4 ± 12.9	34.2 ± 16.8	25.8 ± 13.8
**UICC 2009**				
Ia	5 (7.2%)	12 (29.3%)	0 (0%)	17 (14.3%)
Ib	12 (17.4%)	14 (34.1%)	1 (11.1%)	27 (22.7%
IIa	7 (10.1%)	3 (7.3%)	2 (22.2%)	12 (10.1%)
IIb	34 (49.3%)	7 (17.1%)	6 (66.7%)	47 (39.5%)
III	10 (14.5%)	4 (9.8%)	0 (0%)	14 (11.8%)
IV	1 (1.4%)	1 (2.4%)	0 (0%)	2 (1.7%)
Positive lymph node status*	44 (64.7%)	9 (25.0%)	5 (55.6%)	58 (48.7%)
**Resection margin status**				
R0	62 (89.9%)	38 (95%)	9 (100%)	109 (92.4%)
R1	7 (10.1%)	1 (2.5%)	0 (0%)	8 (6.8%)
Rx	0 (0%)	1 (2.5%)	0 (0%)	1 (0.8%)
Perineural invasion	24 (37.5%)	4 (10.2%)	2 (22.2%)	30 (26.5%)
Lympangiosis	27 (48.2%)	10 (28.6%)	2 (22.2)	39 (39.0%)
ASA scores	2.4 ± 0.6	2.4 ± 0.6	2.4 ± 0.5	2.4 ± 0.5
Data not available, n (%)	6 (8.6%)	3 (7.3%)	—	9 (7.5%)
**Operation**				
Whipple	10 (14.5%)	5 (12.2%)	0 (0%)	15 (12.6%)
PPPD	58 (84.1%)	31 (75.6%)	8 (88.9%)	97 (81.5%)
Ampullary resection	1 (1.4%)	5 (12.2%)	0 (0%)	6 (5.0%)
Others	0 (0%)	0 (0%)	1 (11.1%)	1 (0.8%)
**Op time**				
Mean ± SD	360.5 ± 107.2	350.7 ± 129.6	395.1 ± 44	359.1 ± 116.7
**Hospital stay**				
Mean ± SD	24.3 ± 15.8	23.4 ± 16	25.3 ± 12	24.1 ± 15.5
Adjuvant CTx	8 (11.6%)	3 (7.3%)	0 (0%)	11 (9.2%)

*Patients who received ampullary resection were excluded from analysis, because no lymphadenectomy was performed.

No significant differences were found for demographic data among the groups. The age between patients ranged from 40 to 87 years (Table 1). Most patients were symptomatic at the time of presentation. Obstructive jaundice was the most common symptom in 72.2% of the patients, followed by weight loss (55%), pain (35.3%), and preoperative diabetes (29.4%). Weight loss and obstructive jaundice were more common in patients with the pancreatobiliary subtype, but there was no significant difference compared with the other subtypes. The most frequently performed operation was a pylorus preserving pancreatooduodenectomy (PPPD) in 81.5%, followed by a classic pancreatoduodenectomy in 12.6% of the patients. A transduodenal papillary resection was conducted more often in patients with the intestinal subtype compared with the pancreatobiliary subtype (12.2% versus 1%). With respect to the surgical procedures, statistical significance was found (*p* = 0.003).

The mean tumor size was 25.8 ± 13.8 mm (range: 0–70 mm) and there was no significant difference among the groups. Postoperative length of hospital stay did not differ among the groups (Table 1).

### Tumor markers and tumor stage

All patients showed an elevation of the tumor marker carbohydrate antigen (CA) 19-9 (mean 256.4 U/mL, range: 0–5599 U/mL). The pancreatobiliary subtype also demonstrated an elevation of the carcinoembryonic antigen (CEA) level (mean 7.6 ng/mL, range 0.2–224.6 ng/mL). No significant difference was found among the subtypes regarding CEA and CA 19-9 levels in our cohort (Table 1). The concomitant bilirubin levels were slightly elevated, but not significantly between the groups (Table 1).

The pancreatobiliary subtype is significantly associated with a generally higher tumor stage at the time of presentation compared with the other subtypes (e.g., UICCC stadium IIb [T1 to T3 with concomitant lymph node involvement] in 49.3% of the patients with the pancreatobiliary subtype compared with 17.1% of patients with the intestinal subtype [*p* = 0.004]) (Table 1). We could also demonstrate that the pancreatobiliary subtype has a clearly higher quote of positive lymph nodes in comparison to the intestinal subtype (64.7% versus 25%, *p* < 0.001). Furthermore, the pancreatobiliary subtype showed a higher rate of perineural invasion compared with the intestinal and mixed subtypes (37.5% versus 10.2% and 22.2%, *p = *0.008), respectively. There was no statistically significant difference for resection margin status and lymphangiosis between the groups (Table 1).

### Survival analysis and risk factors

**Figure 1:**
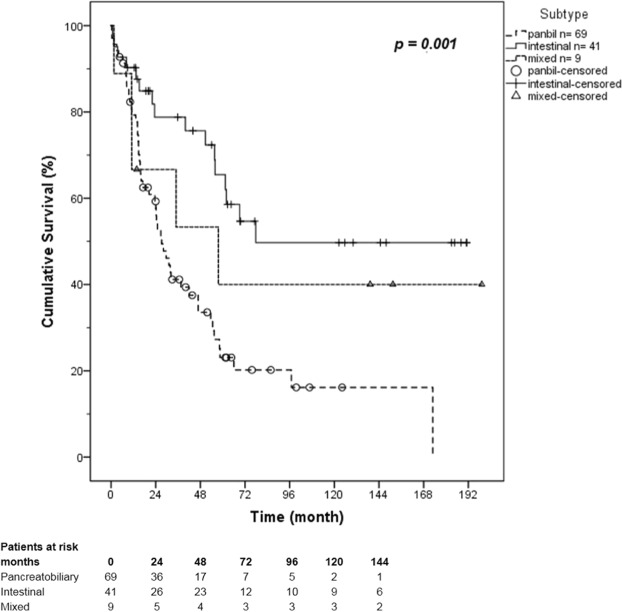
Kaplan-Meier estimation of overall survival of subtypes of ampullary cancer.

The survival analysis of the different subtypes was calculated using the Kaplan-Meier method with log rank test. The mean follow-up time was 48 months (range: 0–199 months). Figure 1 demonstrates the overall survival of the different subtypes. The mean survival time for the pancreatobiliary subtype was 52 months (95% CI: 37–68 months), for the intestinal subtype 115 months (95% CI: 88–142 months), and for the mixed subtype 94 months (95% CI: 35–154 months) (*p* = 0.001) (Table 2). Therefore, the pancreatobiliary subtype shows a significantly poorer overall survival. A significantly worse 5-year survival rate was found for the pancreatobiliary subtype with 27.5% compared with the intestinal subtype with 61% (*p = *0.001). The 5-year survival rate for the mixed subtype was 44.4% (Table 2).

**Table 2:** 5-year survival rate and mean overall survival of patients by subtype.

	Pancreatobiliary	Intestinal	Mixed	All subtypes
**Morphological type**				
5-year survival rate	27.5%	61.0%	44.4%	
Mean overall survival (month) (95% CI)	52.531 (37–68)	115.092 (88–142)	94.694 (35–154)	80.2 (64–96)

5-year survival rate and mean overall survival of all patients divided into subtypes; the mean follow-up time was 48 months (range: 0–199 months).

The univariate analysis using the Kaplan-Meier method with a log-rank test showed that patients with an age older than 65 years had a worse mean overall survival (59.5 month [95% CI 43.2–75.8 month] versus 103.4 month [77.3–129.4 month], *p* = 0.043). Other factors decreasing the mean overall survival were a positive lymph node status (43.2 month [95% CI 27.0–59.3 month] versus 110.7 month [95% CI 87.7–133.7], *p* < 0.001), a R1 resection margin status (16.6 month [95% CI 5.3–27.9 month] versus 81.4 month [95% CI 64.8–97.9], *p* < 0.001), perineural sheet invasion (30.0 month [95% CI 17.4–42.5 month] versus 87.6 month [95% CI 68.7–106.4 month], p = 0.001) and lymphangiosis (60.7 month [95% CI 37.2–84.1 month] versus 94.6 month [95% CI 73.5–115.8 month], *p* = 0.016) (Table 3).

**Table 3:** Risk factors predicting survival.

	Mean overall survival, month (95% CI)	*p*
**Age**		0.043
Age >65 years (n = 67)	59.5 (43.2–75.8)	
Age <65 years (n = 52)	103.4 (77.3–129.4)	
**Diabetes**		0.143
Yes (n = 35)	55.1 (32.6–77.6)	
No (n = 84)	88.0 (68.8–107.3)	
**Jaundice**		0.989
Yes (n = 33)	74.7 (48.9–100.5)	
No (n = 86)	80.6 (61.7–99.4)	
**Weight loss**		0.388
Yes (n = 52)	85.7 (62.3–109.1)	
No (n = 65)	74.1 (53.4–94.8)	
**Pain**		0.114
Yes (n = 42)	64.7 (41.5–87.9)	
No (n = 77)	87.3 (67.0–107.7)	
**Pos**. lymph node status*		<0.001
Yes (n = 57)	43.2 (27.0–59.3)	
No (n = 58)	110.7 (87.7–133.7)	
**Resection margin status**		<0.001
R0 (n = 108)	81.4 (64.8–97.9)	
R1 (n = 8)	16.6 (5.3–27.9)	
**Perineural invasion**		0.001
Yes (n = 30)	30.0 (17.4–42.5)	
No (n = 83)	87.6 (68.7–106.4)	
**Lymphangiosis**		0.016
Yes (n = 39)	60.7 (37.2–84.1)	
No (n = 61)	94.6 (73.5–115.8)	
**Adjuvant CTx**		0.243
Yes (n = 11)	32.7 (21.2–44.3)	
No (n = 108)	81.9 (65.3–98.5)	

Mean overall survival and the 95% (CI) were analyzed by the Kaplan-Meier method using the log-rank test.

A Cox hazard model identified lymph node status (hazard ratio [HR]: 2.259, *p* = 0.007), resection margin status (HR: 5.238, *p* = 0.005) and the pancreatobiliary subtype (HR: 0.45, *p* = 0.022) as independent predictors for poor survival (Table 4).

**Table 4:** Cox regression analysis for risk factors predicting survival.

	*p*	HR	95% CI	
Lymph node involvement	0.007	2.259	1.251	4.081
Resection margin status	0.005	5.238	1.632	16.819
Pancreatobiliary subtype	0.022	0.45	0.227	0.893

The following independent variables were included in the model: Age >65 years, diabetes, jaundice, weight loss, pain, tumor size, positive lymph node status, resection margin status, perineural sheet invasion, lymphangiosis, adjuvant chemotherapy and subtype.

Eleven of 119 patients (9.2%) received an adjuvant chemotherapy. There was no significant difference between the groups with respect to the subtype. The univariate and multivariate analysis showed that the survival rate was not influenced by the adjuvant chemotherapy.

## Discussion

The current study is the largest reported patient cohort (*n* = 119) investigating the prognostic value of subtyping in ampullary cancer. We reported a 5-year survival of 27.5% for patients with the pancreatobiliary subtype and 61% for the intestinal subtype. A small series from Asano *et al*. with 69 patients showed a 5-year survival for the pancreatobiliary and the intestinal subtype of 53.3% and 70.7%, respectively^16^. The prevalence of the pancreatobiliary and the intestinal subtype in our study was 58% and 34.5%, respectively. Compared with the other series, the pancreatobiliary subtype was overrepresented, whereas the intestinal subtype was underrepresented. The overall prevalence of the intestinal type ranged from 27 to 49%, whereas that of the pancreatobiliary type ranged from 21 to 45% in these studies^15–17^. Differences in prevalence could be explained by overrating the MUC-1 expression in the histopathological review in our center. However, the survival data of our groups are consistent with other reports.

The subtype was also found to be an independent survival-predicting risk factor (HR: 0.45, *p* = 0.022) in our study. This also reflects the 5-year (*p = *0.001) and the overall survival (*p* = 0.001) stratified by the subtypes. Other series have also reported a poorer survival of the pancreatobiliary compared with the intestinal subtype^1,18–20^. A limitation of these studies compared with our study is the fact that multiple entities were compared together, such as pancreatic ductal, distal bile duct, ampullary, and duodenal adenocarcinoma. Our study focused only on ampullary cancer to minimize the selection bias of our cohort. A poor prognosis of the pancreatobiliary subtype was also found by our study group in other pancreatic tumors like intraductal papillary mucinous neoplasm^21^.

The poorer prognosis of the pancreatobiliary subtype could probably be explained by a disseminating growth behavior, perineural sheet invasion, and by a distinct desmoplastic stromal reaction^22^. The median overall survival for the pancreaticobiliary subtype was 52 months (95% CI: 37–68 months), which is comparable to resected patients with ductal adenocarcinoma, with an overall survival up to 36 months in selected patients in our center^23^. Localization of the tumor itself could be one of the main reasons for better survival rates, due to the occurrence of early symptoms like obstructive jaundice and pain by occluding the distal bile duct.

Several other studies focused on the evaluation of prognostic factors for ampullary carcinomas. Among these studies, different prognostic factors like T-stage, lymph node involvement, positive surgical margins, perineural invasion, and subtyping were reported^1,3,4,24,25^. In our study, the frequency of lymph node involvement was significantly higher in patients with pancreatobiliary subtype (64.7% versus 25% intestinal subtype, *p* < 0.001). Based on the results of the univariate analysis, positive lymph node status was identified as the survival-predicting risk factor. Also the rate of perineural invasion was significantly higher in patients with pancreatobiliary subtype. Even it was not an independent prognostic factor for poor survival in the Cox regression analysis, we found a worse survival of patients with positive perineural invasion in the univariate analysis. In a meta-analysis by Luchini *et al*. perineural invasion was strongly associated with a poorer prognosis in patients with ampullary cancer^26^. This behavior is based on the origin of the epithelium, which arises from the distal bile duct, distal pancreatic duct, or the common bile duct of the ampulla. Because of these characteristics, tumors with the pancreatobiliary subtype have more aggressive growth and present at a more advanced T-stage than the intestinal subtype. The better prognosis of the intestinal subtype could be explained by its precursor lesions, which arise from preexisting adenomas following the adenoma-dysplasia-adenocarcinoma sequence^27^. For this reason, this intestinal subtype is less aggressive compared with the pancreatobiliary subtype. For patients with the mixed-type differentiation, an intermediate prognosis can be stated. This is confirmed by further data^28^.

In our cohort, there were six patients with the intestinal subtype who received a transduodenal ampullary resection. In two of the six patients the intraoperative pathohistological examination showed a carcinoma. In one patient, a PPPD was performed and one patient was unfit for an extended surgery. Four of six patients had a pTis or pT1 tumor stage. As an individual therapeutic approach, active surveillance in these five of six patients was recommended. There was no tumor-related death in this cohort. Nevertheless, we cannot conclude that a parenchyma-sparing resection should be performed in patients with the intestinal subtype. Intraoperative histopathologic subtype determination is not possible. If the final examination of the surgical specimen detects the intestinal subtype and there are no radiological signs of lymph node involvement in the preoperative staging CT/MRI surveillance can be considered for high-risk patients after a parenchyma-sparing resection.

Two patients with UICC stage IV disease were identified in our study population. In these two patients’ para-aortic lymph node metastases, which are classified as distant metastases (M1), were detected in the examination of the surgical specimen. In a recent study of our center the survival of resected patients with para-aortic lymph node metastases was comparable with advanced pN + stages and one-third of the patients may expect longer survival after radical resection of pancreatic ductal adenocarcinoma^29^. Further studies identified elevated CA 19-9 and CEA levels as a relevant prognostic factor^4,30^. Kim *et al*. showed that elevated CEA is an important predictor of recurrence in ampullary cancer^4^. Another study by Okano *et al*. showed that elevated CA 19-9 levels were prognostic factors in patients with the pancreatobiliary subtype. In contrast, our analysis showed only slightly elevated CEA levels in patients with the pancreatobiliary subtype, whereas all subtypes have shown increased levels of the tumor marker CA 19-9. The effect of elevated tumor markers in patients with ampullary cancer has to be critically discussed, as most patients showed an obstructive jaundice with elevated bilirubin levels at the time of presentation. CA 19-9 and CEA undergo biliary excretion, and serum levels may be artificially increased due to liver cirrhosis and benign inflammatory or cholestatic diseases of the pancreatobiliary tract^31–34^.

Future directions of subtyping ampullary cancer could be, in addition to predicting survival, the determination of therapy regimes. Retrospective studies suggest a potential benefit in administering adjuvant chemotherapy in patients with lymph node involvement^35,36^. These findings were confirmed by a retrospective national cohort study with 4,190 patients by Nassour *et al*. The use of adjuvant therapy in ampullary cancer was associated with significantly improved overall survival^37^. Currently, there is no suggestion for the use of adjuvant chemotherapy.

Neoptolemos *et al*. found in the randomized multicenter ESPAC-3 study no difference in survival relating to the adjuvant use of gemcitabine or 5-FU versus observation in the subgroup analysis of patients with ampullary cancer of the intestinal and pancreatobiliary subtype^38^. The results of the ESPAC-3 study were underlined by a recent meta-analysis, in which no associated survival benefit to adjuvant therapy was found^7^. Eleven of 119 patients (9.2%) received an adjuvant therapy in our study and the analysis showed that there was no survival benefit in this small patient cohort. Adjuvant therapy should be evaluated in prospective clinical trials in a larger cohort patients with the pancreatobiliary subtype. It is also assumed that the different subtypes may show variable responses to different chemotherapy regimens. Thus, gemcitabine-based therapy, for example may be favorable as there is no level one evidence that this is the case for the pancreatobiliary subtype and fluoropyrimidine-based regimes for the intestinal subtype^39,40^.

## Conclusion

The pancreatobiliary subtype is associated with poor prognosis and a higher rate of lymph node metastasis, whereas the intestinal subtype shows an excellent prognosis. Prospective studies are recommended to evaluate the effect of subtype-stratified adjuvant chemotherapy.

## Patients and Methods

Within this analysis we evaluated 170 patients with ampullary cancer, who underwent resection between 1999 and 2016 at our department. A total of 119 patients with complete immunohistochemical subtype analysis were included in the study. Demographic characteristics, clinical symptoms, laboratory parameters, intra- and postoperative parameters, histopathological characteristics, and a long-term follow-up until 2017 were determined. Patients with other types of ampullary cancer like adenosquamous cell carcinomas, neuroendocrine tumors, and mucinous adenocarcinomas were excluded from this study. Tumor stage was defined according to the TNM classification published by the UICC (Union of International Cancer Control) in 2010^41^.

### Ethical approval and informed consent

All procedures performed in studies involving human participants were in accordance with the ethical standards of the institutional research committee and with the 1964 Helsinki declaration and its later amendments or comparable ethical standards. Informed consent was obtained from all individual participants included in the study.

In accordance with the guidelines for research on human subjects, approval was obtained from the local ethics committee at the Technische Universität Dresden (decision number EK 59032007). The ethics committee is registered as institutional review board (IRB) at the Office for Human Research Protections (OHRP) (registration number (IRB00001473 and IORG0001076).

### Pathology review

All available slides of the 119 patients included in this study were re-evaluated by two pathologists. Tumors were classified as intestinal or pancreaticobiliary subtypes according to histomorphology and immunohistochemical expression profile using hematoxylin & eosin and immunohistochemical expression analyses of CK7, CK20, CDX2, und MUC1 according to standard protocols, according to World Health Organization criteria. The pancreaticobiliary subtype expressed CK7 and MUC1, whereas CK20 and CDX2 were not expressed. The intestinal subtype only expressed CK20 and CDX2. CK7 and MUC1 were negative. Tumors with indistinct morphological and/or immunhistochemical characteristics were classified as a mixed type.

### Statistical analysis

A statistical analysis was performed using IBM SPSS Statistics (Version 24, SPSS Inc, Chicago, IL). In analyzing the descriptive data, the mean, chi-square test, and analysis of variance (ANOVA) were used. Corrections for multiple comparisons were performed using the Bonferroni method. For testing independent predictors for poor overall survival, a Cox hazard model using survival as dependent variable with stepwise backward eliminations based on the likelihood ratios was employed. The following independent variables were included in the model: Age >65 years, diabetes, jaundice, weight loss, pain, tumor size, positive lymph node status, resection margin status, perineural sheet invasion, lymphangiosis, adjuvant chemotherapy and subtype. Independent predictors were expressed as hazard ratio (HR) and 95% % confidence interval (CI). *P* values less than 0.05 were considered to be statistically significant. Survival probability and the 95% (CI) were analyzed by the Kaplan-Meier method using the log-rank test and graphed by Kaplan-Meier curves. The survival time was calculated from the date of operation until death or last follow-up.
